# Parasitoid Complex Associated With the Overwintering Generation of *Swammerdamia pyrella* (Lepidoptera: Yponomeutidae) in Poland

**DOI:** 10.1093/jisesa/ieu126

**Published:** 2014-01-01

**Authors:** Edyta Górska-Drabik, Izabela Kot, Katarzyna Golan, Katarzyna Kmieć

**Affiliations:** ^1^ Department of Entomology, University of Life Sciences in Lublin, Leszczyńskiego 7, 20-069 Lublin, Poland

**Keywords:** Ichneumonidae, Chalcidoidea, pest, natural regulation

## Abstract

The study was conducted on fruit trees where bands of corrugated cardboard were attached around the trunks of the trees, which were used to catch the larvae of overwintering generation of the rufous-tipped swammerdamia moth, *Swammerdamia pyrella* (Villers) (Lepidoptera: Yponomeutidae). Twenty-five species of parasitic Hymenoptera have been described from *S. pyrella* in Poland including the report in this article of seven species belonging to the family of Ichneumonidae (three species) and superfamily Chalcidoidea (four species). The parasitoids *Gelis agilis* F. (Ichneumonidae), *Chrysocharis aquilegiae* (Erdös) (Eulophidae), *Catolaccus ater* (Ratzeburg) (Pteromalidae), and *Eupelmus urozonus* (Dalman) (Eupelmidae) had not been reported from the host before. *Triclistus pallipes* Holmgren (Ichneumonidae), *Dibrachys cavus* Walker (Pteromalidae) had the greatest effect on the natural regulation of *S. pyrella* population. Parasitization for the wintering cocoons of *S. pyrella* changed each year, but it was high throughout the study. The contribution of secondary parasitoids was much higher than primary parasitoids.

The rufous-tipped swammerdamia moth, *Swammerdamia pyrella* (Villers) (Lepidoptera: Yponomeutidae), is a common species throughout Europe, and occurs mostly in dispersed populations ( Baraniak 1992 ). In the 1970s, it was a serious pest in many orchards of western Poland, especially those that were treated with insecticides ( Kadłubowski and Szmyt 1985 ). Actually, *S. pyrella* is not present in the orchards with chemical pest control; therefore, interest in this species is minimal, resulting in a low number of reports about its occurrence in recent literature. However, a trend to reduce pesticides in crops of many plants creates new possibilities for the development of this species. The research indicates that *S. pyrella* successfully found its own niches, which are fruit orchards with limited pest management ( Górska-Drabik 2003 ) and newly planted apple orchards ( Velcheva 2009 ). Therefore, it is important to understand the community of organisms that naturally reduce the number of *S. pyrella* . An assessment of their role makes it possible to evaluate the real threat posed by this species.

Larvae of *S. pyrella* feed on *Malus domestica* Borkh. ( Buszko 1991 ), *Cerasus avium* (L.), *Prunus* spp. ( Kadłubowski and Szmyt 1985 ), *Pirus* spp., *Sorbus* spp., and *Crataegus* spp. ( Maciesiak and Boczek 1983 ). In the beginning, the larva feeds within a leaf, producing mine, then they leave the mines and feed on the surface as an exophagous species. It eats the epidermis and the flesh, leaving the nerves and the bottom skin. In addition, it ties the leaf edge with its web so that forms a “boat.” The leaves become brown and then dry out. The pupation takes place in a white spindle-like cocoon with thread-like insets on the leaves (first generation) and in bark crevices, on the trunks (second generation) ( Maciesiak and Boczek 1983 , Baraniak 1992 ).

So far, little information is available on the species structure of parasitoid community associated with *S. pyrella* , especially with the second generation of larvae overwintering in bark crevices of trees ( Kadłubowski and Szmyt 1985 ). This article identifies the Hymenoptera parasitoid complex of *S. pyrella* in eastern Poland and defines the parasitization level of second-generation caterpillars.

## Materials and Methods

### 

#### Study Area

The study was conducted between 2001 and 2003 in three sites located in Lublin and its vicinity (SE Poland).

Site 1—a monastery garden in the centre of Lublin (UTM - FB 08; 51.247° N, 22.560° E). Ten apple, pear, and plum trees grown together with vegetables and ornamental plants were several decades old with large, branchy crowns. No treatment for plant protection was applied there. The garden was separated from a housing estate and a shopping center by a brick wall.

Site 2—a fruit orchard in Marynin (UTM - EB 98, 51.214° N, 22.427° E), 16 km away from Lublin. Apple, pear, and plum trees growing in an area of about 0.05 ha were 30–40-yr-old. They had not been sprayed, pruned, or fertilized.

Site 3—an apple orchard located in Leonów (UTM - FB 19; 51.340° N, 22.635° E) 14 km away from Lublin. It was a 5-ha orchard planted in 1986, and the main varieties included Cortland, Idared, and Golden Delicious. It was a commercial orchard with semi-dwarf plantings until 1993. Since then, no chemical treatments or fertilization has been applied.

#### Traps

In each site, 25-cm-wide stripes of corrugated cardboard were attached around the trunks of the trees, 30–60 cm above the ground. In sites 1 and 2, all the trees were taken into account, respectively, 10 and 13, whereas in site 3, 45 trees were randomly selected. The bands were attached around the trunks in May and removed in October. In total, 204 bands were used over the 3 yr of study. In the laboratory of the Department of Entomology (University of Life Sciences in Lublin), the collected traps were examined. The cardboard fragments with *S. pyrella* cocoons were cut out and they were placed individually in test tubes. The material prepared in this way was placed in an outside insectarium with the aim of exposing them to low temperatures. In February, the material was transferred into the laboratory and stored at room temperature (20–23°C) until the emergence of the adults (imago of parasitoids or moths). Then they were killed by ethyl acetate, prepared, identified, and counted.

The recorded parasitoids were identified using the keys of Kasparyan (1981) and Trjapitzin (1978) . The nomenclature of parasitoids was verified after Kaźmierczak (2004) and Noyes (2007) . The obtained specimens of Hymenoptera were deposited in the Department of Entomology, University of Life Sciences in Lublin (Poland).

#### Data Analysis

The results into the degree of parasitization of the *S. pyrella* cocoons were statistically analyzed using a one-sided significance test between the two indicators of the structure (available in Statistica 6.0, StatSoft), at statistical significance of *P* < 0.05.

## Results

Twenty-five species of parasitic Hymenoptera have been described from *S. pyrella* in Poland including the literature data as well as species obtained as a result of own studies ( Table 1 ). Rearing 303 cocoons of *S. pyrella* resulted in obtaining 112 imagines of parasitic Hymenoptera ( Tables 2 and 3 ), 8 of which were not identifiable species because they were damaged. They represented seven species grouped into four families: Ichneumonidae (three species), Eulophidae (one species), Pteromalidae (two species), and Eupelmidae (one species). Parasitoids as *Gelis agilis* F., *Chrysocharis aquilegiae* (Erdös), *Catolaccus ater* (Ratzeburg), and *Eupelmus urozonus* (Dalman) had not been reported from the host before.

**Table 1.** List of parasitoids recorded from *S. pyrella*

Superfamilies	Families	Subfamilies	Species	References	Own research
Ichneumonoidea	Ichneumonidae	Pimplinae (=Ephialtinae)	*Itoplectis maculator*	Kadłubowski and Szmyt (1985)	–
			*Itoplectis alternans*	Górska-Drabik (2003)	–
		Gelinae (=Cryptinae)	*Gelis integer*	Kadłubowski and Szmyt (1985)	–
			*Gelis areator*	Górska-Drabik (2003)	–
			*Gelis agilis*	–	+N
			*Hemiteles decipiens*	Kadłubowski and Szmyt (1985)	–
			*Hemiteles chionops*	Kadłubowski and Szmyt (1985)	–
		Metopiinae	*Triclistus pallipes*	Kadłubowski and Szmyt (1985)	+
			*Triclistus pygmaeus*	Napiórkowska-Kowalik and Winiarska (2001)	–
			*Triclistus spiracularis*	Górska-Drabik (2003)	–
		Ichneumoninae	*Phaeogenes impiger*	Kadłubowski and Szmyt (1985)	–
			*Phaeogenes minimus*	Kadłubowski and Szmyt (1985)	–
			*Herpestomus brunnicornis*	Napiórkowska-Kowalik and Winiarska (2001)	+
			*Herpestomus nasutus*	Górska-Drabik (2003)	–
	Braconidae	Microgasterinae	*Apanteles xanthostigma*	Kadłubowski and Szmyt (1985) and	–
				Górska-Drabik (2003)	
			*Apanteles longicauda*	Górska-Drabik (2003)	–
		Agathidinae	*Earinus tuberculatus*	Górska-Drabik (2003)	–
Chalcidoidea	Eulophidae	Entedoninae	*Chrysocharis aquilegiae*	–	+N
		Eulophinae	*Cirrospilus pictus*	Kadłubowski and Szmyt (1985)	–
			*Sympiesis acalle*	Kadłubowski and Szmyt (1985)	–
			*Elasmus albipennis*	Kadłubowski and Szmyt (1985)	–
		Tetrastichinae	*Sphenolepis * ? *oreophilus*	Kadłubowski and Szmyt (1985)	–
	Pteromalidae	Pteromalinae	*Dibrachys cavus*	Kadłubowski and Szmyt (1985)	+
			*Catolaccus ater*	–	+N
	Eupelmidae	Eupelminae	*Eupelmus urozonus*	–	+N
Total			25	21	7 (4N)

*N* first record from *S. pyrella* .

**Table 2.** *S. pyrella* cocoons collected in 2001–2003 and factors limited their number

Year	Number of cocoons			
	In total	Parasitized	Died of unknown causes	From which moths were obtained
2001	152	64	40	48
2002	96	13	44	39
2003	55	7	29	19
Total	303	84	113	106

**Table 3.** Species of Hymenoptera recorded from the cocoons of *S. pyrella* in 2001–2003

Species	Number of individuals			Total	Contribution to the parasitoid complex (%)	Contribution to parasitization (%)
	2001	2002	2003			
Parasitoids						
*T. pallipes*	3	1	–	4	30.8	1.3
*H. brunnicornis*	2	–	–	2	15.4	0.7
*Ch. aquilegiae*	–	–	15 (2)	15 (2)	15.4	0.7
*D. cavus*	–	18 (4)	–	18 (4)	30.8	1.3
*E. urozonus*	–	–	1	1	7.7	0.3
Sum	5	19 (5)	16 (3)	40 (13)	100	4.3
Hiperparasitoids						
*G. agilis*	58	8	–	66	92.9	23.4
*Gelis* sp.	–	–	4	4	5.6	
*C. ater*	2 (1)	–	–	2 (1)	1.4	
Sum	60 (59)	8	4	72 (71)	100	23.4
Total	65 (64)	27 (13)	20 (7)	112 (84)	–	27.7

The species composition of the parasitoid complex was changing each year. There was no consistent recording of the species within the study period. Only two species, namely *G. agilis* and *Triclistus pallipes* Holmgren, occurred in two successive years of studies, whereas the others were observed only in 1 yr. Among the recorded parasitoids, the species *Herpestomus brunnicornis* (Gravenhorst), *E. urozonus* , and *C. ater* were rarely found and in small numbers. The greatest number of parasitoids was observed in 2001 ( Table 3 ).

The most numerous trophic groups were hyperparasitoids, involving only two species: *G. agilis* and *C. ater* . Four specimens have been identified only as genus *Gelis* ( Table 3 ).

Of all the obtained parasitoids, 15.5% were primary parasitoids of *S. pyrella* , whereas the others fell within the unknown species of parasitic Hymenoptera from which hyperparasitoids were recorded. *T. pallipes* and *Dibrachys cavus* (Walker) were the most abundant species in the complex of primary parasitoids and they represented the greatest number of parasitized *S. pyrella* cocoons ( Table 3 ). The contribution of *Ch. aquilegiae* , *D. cavus* , and *E. urozonus* in parasitization is not as obvious because they can be both primary and secondary parasites ( Table 4 ). *G. agilis* represented 94% of the community of secondary parasitoids.

**Table 4.** Parasitoids recorded from the cocoons of *S. pyrella* and their status

Species	Status ^a^					
	Primary	Secondary	Endoparasite	Ectoparasite	Solitary	Gregarious
*I. maculator*	+		+ (of pupae)		+	
*I. alternans*	+		+ (of pupae)		+	
*G. integer*	+	+		+	+	
*G. areator*		+		+	+	
*G. agilis*		+		+	+	
*H. decipiens*	+ ?	+ ?		+	+	
*H. chionops*	+ ?	+ ?		+	+	
*T. pallipes*	+		+		+	
*T. pygmaeus*	+		+		+	
*T. spiracularis*	+		+		+	
*P. impiger*	+		+		+	
*P. minimus*	+		+		+	
*H. brunnicornis*	+		+		+	
*H. nasutus*	+		+		+	
*A. xanthostigma*	+		+		+	
*A. longicauda*	+		+		+	
*E. tuberculatus*	+		+		+	
*Ch. aquilegiae*	+	+		+	+	
*C. pictus*	+			+	+	
*S. acalle*	+	+		+	+	
*E. albipennis*		+		+	+	
*S.* *oreophilus*	+ ?					
*D. cavus*	+	+	+			+
*C. ater*		+		+	+	
*E. urozonus*	+	+		+	+	

^a^ The status of the obtained parasitoid species is given according to de Graham (1969) , Trjapitzin (1978) , Górny (1979) , Zajančkauskas et al. (1979) , Kasparyan (1981) , Noyes (2003) , Stojanović and Markocić (2005) , Mafi and Ohbayashi (2010)

**Fig. 1.**
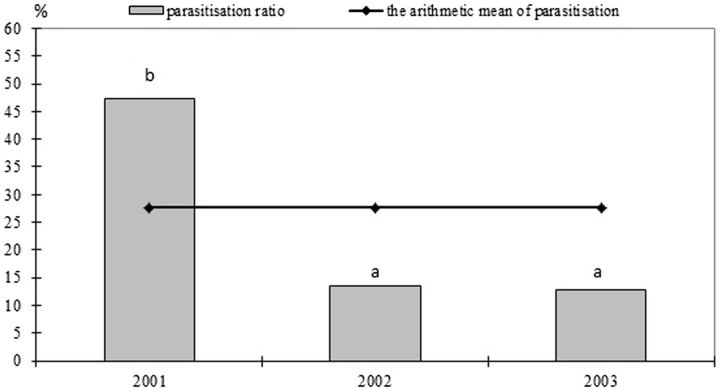
Parasitization level of *S. pyrella* cocoons in 2001–2003. Histograms with the same letters above do not differ significantly ( *P* < 0.05).

In total, 30.4% of the wintering cocoons of *S. pyrella* were parasitized by the Hymenoptera species throughout the study. In 2001, it was at its highest 47.4%, whereas in 2002 and 2003 the level of parasitization was respectively at 13.5 and 12.7% ( Fig. 1 ). The percentage of parasitized cocoons in 2001 was significantly higher than in 2002 as well as in 2003 ( *P* < 0.05).

## Discussion

The aim of this study was to document the parasitoid complex of *S. pyrella* in Poland. Using published reports and own studies showed that the parasitoids community is represented by 25 species grouped into two families, Ichneumonidae and Braconidae, as well as the superfamily of Chalcidoidea. There is no information about other natural enemies of *S. pyrella* both in Poland and in Europe.

In Poland, the parasitoid community of *S. pyrella* was studied by Kadłubowski and Szmyt (1985) . They recorded 13 parasitoid species including seven belonging to the family of Ichneumonidae, one species from the family Braconidae, and five species from the superfamily Chalcidoidea. Later research increased this list with two species from Ichneumonidae ( Napiórkowska-Kowalik and Winiarska 2001 ). Górska-Drabik (2003) showed another four species from Ichneumonidae and two species from Braconidae, which were new to this host. The results presented in this article enlarged the list of the parasitoids complex of *S. pyrella* with three species belonging to the superfamily of Chalcidoidea and one species from the family of Ichneumonidae.

Hyperparasitoids were the most numerous trophic group, and among them, *G. agilis* was the species that occurred in the greatest number. Species from the genus *Gelis* Thnbg. are parasites of Ichneumonidae and Braconidae ( Zajančkauskas et al. 1979 , Kasparyan 1981 ). Piekarska-Boniecka (1997) described this species as hyperparasitoid of *Archips rosanus* L. while Sawoniewicz and Buszko (1994) gave another species— *Bucculatrix nigricomella* Zeller as its host. *C. ater* is another recorded hyperparasitoid species and is stated as a parasitoid of the Braconidae family mainly for *Apanteles* spp. ( Trjapitzin 1978 ). It was also reported as hyperparasitoid of Gracillariidae ( Stojanović and Markocić 2005 ).

Numerous hyperparasitoids obtained in this study (70 individuals) could be the cause of an absence of primary parasitoids especially from the Braconidae family. Such species as *Apanteles xanthostigma* (Haliday), *Apanteles longicauda* Wesmael, *Earinus tuberculatus* (Wesmael), and *Oncophanes lanceolator* Ness, belonging to this family were parasitoids of *S. pyrella* ( Zajančkauskas et al. 1979 , Kadłubowski and Szmyt 1985 , Górska-Drabik 2003 ). *A. xanthostigma* was recorded in Górska-Drabik (2003) study as the most effective parasitoids from the family of Braconidae—it is parasitized ∼10% of *S. pyrella* larvae and pupae. An important role of genus Apanteles was also emphasized by Lucchese (1941) . In the orchards of central Italy, it parasitized 80% of the population of *S. pyrella* larvae.

The group of primary parasitoids included five species although the status of three of them ( *Chrysocharis pentheus* (Walker), *E. urozonus* , and *D. cavus* ) is not clear. According to Trjapitzin (1978) , *D. cavus* , from the family of Pteromalidae, can be a primary or secondary polyphagous parasitoid of many species of Lepidoptera as well as some Diptera and Hymenoptera. It was reported from *Yponomeuta malinellus* (Zeller) (Yponomeutidae) as well as other families’ of moths like Lymantriidae, Choreutidae, and Tortricidae ( Zajančkauskas et al. 1979 ). Winiarska and Anasiewicz (1989) enumerated this species as a parasitoid of the moths’ larvae wintering under the bark of apple trees. It was also reported from the larvae of second generation of *S. pyrella* ( Kadłubowski and Szmyt 1985 ). However, *E. urozonus* obtained from *S. pyrella* can be a primary or secondary parasitoid. It is a part of the parasitoid complex of Coleoptera, Lepidoptera, and Diptera ( Górny 1979 ). Reported from *Callisto denticulella* (Thunberg) ( Górska-Drabik and Napiórkowska-Kowalik 2009 ) and another species of the family Gracillariidae ( Grabenweger 2004 ). *Ch. aquilegiae* is also a new parasitoid of *S. pyrella* , which has not been reported so far. This species is known as a parasitoid of different species of Diptera and Coleoptera, mainly however of moths ( Trjapitzin 1978 , Szczepański 1983 , Hansson 1985 , Adachi 1998 , Jenser et al. 1999 , Noyes 2007 ). It can also be a secondary parasitoid ( Trjapitzin 1978 ).

*T.* *pallipes* had been earlier reported by Kadłubowski and Szmyt (1985) , as *Triclistus pallidipes* Dalla Torre from cocoons of first generation *S. pyrella* . This species is an endoparasite of Tortricidae, Phycitidae, and other moths ( Kasparyan 1981 , Jonaitis 2000 ). It was reported from *Strophedra weirana* Douglas and *Rhopobota naevana* (Hübner), both from Tortricidae ( Tolkanitz 1987 ).

*H* *.* *brunnicornis* had been earlier reported as the parasitoid of *S. pyrella* ( Napiórkowska-Kowalik and Winiarska 2001 ). Więckowski and Więckowska (1961) , Kot (1964) , and Gençer (2003) also reported this species in parasitoid complex of *Y. malinellus* belonging also to the family Yponomeutidae. *H. brunnicornis* is mentioned as endoparasitoid of many families of moths: Yponomeutidae, Plutellidae, Tortricidae ( Miczulski and Koślińska 1976 , Kasparyan 1981 ).

The available studies include very little information on the natural regulation of *S. pyrella* . The greatest role in limiting its population is parasitoids from the families of Ichneumonidae and Braconidae ( Kadłubowski and Szmyt 1985 , Napiórkowska-Kowalik and Winiarska 1999 , Górska-Drabik 2003 ). On the other hand, the authors’ own studies indicated that *T. pallipes* , *D. cavus* , and *G. agilis* were the most abundant. A sparse occurrence of *H. brunnicornis* was observed in parasitoid complex of *S. pyrella* (only two specimens). While Napiórkowska-Kowalik and Winiarska (1999) indicated an 18% of parasitization of *S. pyrella* population by this species.

In total, more than 30% of *S. pyrella* cocoons were parasitized throughout the study—in successive years it ranged from 13 to 48%. However, it has been reported that parasitization of this species larvae was lower and amounted to over 16% ( Górska-Drabik 2003 ).
